# Transitional implants in computer‐assisted implant surgery and fixed complete‐arch provisionalization: A retrospective case series

**DOI:** 10.1111/cid.13396

**Published:** 2024-11-17

**Authors:** Ignacio Pedrinaci, Betty Ben Dor, Dominique Rousson, Alejandro Lanis, Javier Sanz‐Esporrin, Kevser Pala, German O. Gallucci, Adam Hamilton

**Affiliations:** ^1^ Department of Restorative Dentistry and Biomaterials Science, Harvard School of Dental Medicine Harvard University Boston Massachusetts USA; ^2^ Section of Graduate Periodontology, School of Dentistry University Complutense Madrid Spain; ^3^ DMD Candidate Harvard School of Dental Medicine Boston Massachusetts USA; ^4^ ETEP (Etiology and Therapy of Periodontal and Peri‐implant Diseases) Research Group University Complutense Madrid Spain; ^5^ Division of Oral Restorative and Rehabilitative Sciences University of Western Australia Perth Western Australia Australia

**Keywords:** edentulous maxilla, edentulous mandible, fixed implant prostheses, retrospective, survival rate, full‐arch

## Abstract

**Introduction:**

Using mini implants as transitional implants (TIs) for complete arch implant‐supported rehabilitations may overcome limitations associated with mucosa‐supported surgical guides and facilitate immediate fixed provisionalization. This study aimed to assess the success of TIs in supporting surgical guides for implant placement and fixed provisional prostheses.

**Methods:**

Patients who received TIs between 2012 and 2023 for a complete arch implant‐supported prosthesis were evaluated retrospectively. Patient demographic data, TI functionality in supporting a surgical guide and supporting a complete arch provisional prosthesis, and dates of TI placement and regular implant placement were collected. Descriptive statistics were used to determine the survival rate and success rate for TIs.

**Results:**

Twenty‐six patients, 35 jaws, 136 TIs, and 216 regular implants were included. The survival rate of TIs was 74.26%; however, the use of TIs yielded success in 97% of jaws for supporting a surgical guide and a fixed complete‐arch provisional prosthesis throughout the complete provisional phase. An average of 4 TIs per maxilla and 3 TIs per mandible supported surgical guides. Thirty‐five provisional prostheses were placed on an average of 4 TIs in the maxilla and 3 TIs in the mandible. Thirty‐four provisional prostheses were successfully supported by TIs and regular implants until final restoration delivery. The survival of regular implants placed in conjunction with the use of TIs was 98%.

**Conclusions:**

Using TIs to support a surgical guide and provisional prosthesis may be a predictable approach with a high success rate. All surgical guides planned to be supported on TIs were successful. Despite premature loss or replacement of TIs, this approach was able to support most provisional prostheses until the regular implants could be loaded.

**Summary Box**

**What is known**

- Mucosa or bone‐supported implant surgical guides are less accurate than tooth or implant‐supported guides.
- Patients undergoing extensive bone reconstructive procedures need undisturbed extended healing time, limiting the use of removable prostheses.
- Immediate implant loading is not always feasible in complete‐arch rehabilitation, particularly alongside large bone reconstructive procedures.

**What this study adds**

- TIs provide stable support for surgical guides during implant placement.
- TIs offer patients a fixed provisional prosthesis where regular implants are placed in conjunction with guided bone regeneration, or where immediate loading is considered high‐risk
- The survival rate and success of using TIs for guided implant surgery and fixed provisional restorations are presented in this study

## 1 INTRODUCTION

Edentulous patients constitute a substantial portion of the current patient demographic.^1^ Different treatment options, such as complete dentures and implant‐supported or retained prostheses, are available to restore function and esthetics in edentulous patients.^2^

According to a recent consensus report, implant‐supported prostheses increased patient satisfaction with stability and comfort compared to complete dentures.^3^ Furthermore, complete implant‐supported fixed dental prostheses may be recommended over implant‐retained overdentures when clinically indicated to achieve the highest levels of stability, retention, and comfort.^3^ Complete arch rehabilitations with implant‐supported fixed prostheses are a well‐established treatment approach.^4^ A patient‐specific and detailed biorestorative^5^ treatment plan must be generated to achieve predictable treatment outcomes and improve the long‐term success of implant‐supported prostheses.^6^ The advancement of digital technologies allows for treatment planning that considers site‐specific anatomy and the intended prosthetic design.^7^ The surgical and prosthetic strategies can be pre‐assessed in a virtual environment, and surgical implant guides can be manufactured based on the digital treatment plan to perform a static computer‐assisted implant surgery (s‐CAIS).^8^

Using a surgical implant guide may increases the accuracy and safety of the implant placement procedure, reduces chair time, and allows for less invasive surgical interventions, such as a flapless implant placement.^9^, ^10^ Complete edentulism presents a challenge for s‐CAIS, as the absence of teeth or existing implants complicates the fixed positioning of the surgical guide. Bone‐supported guides were shown to result in increased patient morbidity due to the need for greater ridge exposure and, similar to mucosal‐supported guides, less accurate implant placement compared to tooth‐supported guides.^7^, ^11^, ^12^, ^13^, ^14^

An alternative treatment approach utilizing mini implants as transitional implants (TIs) to support and position a surgical guide in completely edentulous patients during s‐CAIS can assist in overcoming the limitations of bone or mucosa‐supported templates.^15^ When considering complete arch implant restorations, patient comfort and satisfaction increase when an immediate loading protocol is used.^16^ However, immediate loading of regular implants is not always possible; contraindications for immediate implant loading may include a lack of primary stability or the application of extensive guided bone regeneration procedures.^17^, ^18^, ^19^ The use of TIs in this technique not only enhances the precision of s‐CAIS but can also support provisional prostheses if immediate loading of the implants is not preferred or is contraindicated.^15^, ^20^

Further development and modification of the original published TI protocol allows for a diagnostic tooth setup to plan the position of the TI and regular implants simultaneously, producing a stackable surgical guide to place both types of implants. This approach can reduce the number of surgical interventions to one appointment. A mucosa‐supported stackable guide is first used to place the TIs. They are then picked up intraorally with a second surgical guide, and regular implants are placed fully guided with an s‐CAIS protocol. Since the surgical guide is implant‐supported and screw‐retained, full flaps can be raised without affecting the accuracy of the guided surgery which facilitates improved visualization, access for surgical irrigation as well as concomitant regenerative procedures. Subsequently, the TIs can support a prefabricated provisional.^15^, ^20^

Therefore, the present study aimed to assess the clinical utility of TIs in supporting the surgical guide during s‐CAIS and their ability to support a provisional prosthesis until the delivery of the final implant‐supported restoration.

## 2 MATERIALS AND METHODS

### 2.1 Experimental design/sample

This study was designed as a retrospective case series and was conducted per the Strengthening the Reporting of Observational Studies in Epidemiology (STROBE) guidelines.^21^, ^22^ Patient and implant‐related data were obtained from the electronic health records of patients treated at Harvard School of Dental Medicine from 2012 to 2023.

### 2.2 Ethical approval

Ethical approval was obtained by the Office of Human Research Administration at Harvard Medical School (IRB17‐1026).

### 2.3 Eligibility criteria and recruitment

The following inclusion criteria were used to define eligibility for patients in the database: (1) age greater than 18 years old; (2) complete edentulism; (3) transitional (mini) implants placed to support provisional prostheses between 03/2012 and 05/2023. The following exclusion criteria were defined: (1) TIs were planned for any other purpose (e.g., to support an overdenture for final restoration); (2) treatment with final implant‐supported fixed complete arch prosthesis had not been completed; (3) immediate loading of the prosthesis on the regular implants.

### 2.4 Treatment concept

**FIGURE 1:**
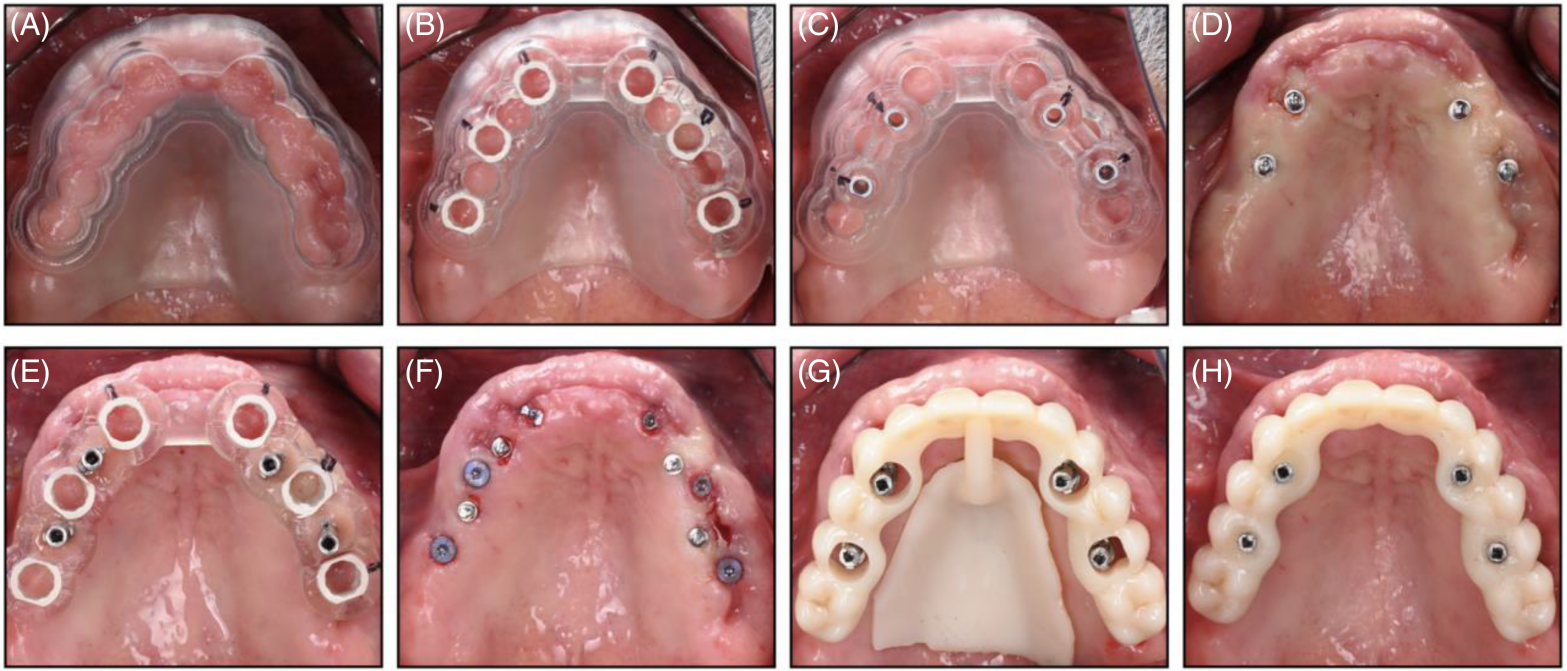
Clinical example of a flapless maxillary case: (A) Mucosa‐supported stackable guide; (B) Surgical guide for regular implant surgery, shown here tried in on top of (A) stackable guide; (C) Surgical guide for TI placement, shown here tried in on top of (A) stackable guide; (D) TI position after guided placement; (E) Surgical guide for regular implant placement supported on TIs; (F) Regular implant position after guided placement; (G) Position of provisional prosthesis; (H) Provisional prosthesis retained on TIs.

**FIGURE 2:**
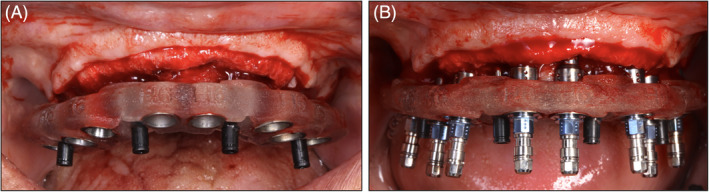
Clinical example of a TI‐ supported guide that allows for a flap to be raised for bone reconstructive purposes. Note that this is one of the main benefits compared to a mucosa supported guide where a flap can’t be raised. (A) TI‐supported guide (B) Regular implant placement on a TI‐supported surgical guide.

**FIGURE 3:**
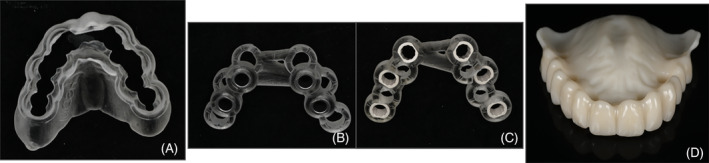
(A) Mucosa‐supported stackable guide used to support (B) and (C) for implant surgery; (B) Surgical guide for TI placement; (C) Surgical guide for regular implant placement; (D) Provisional prosthesis with holes to be picked up on TIs.

**FIGURE 4:**
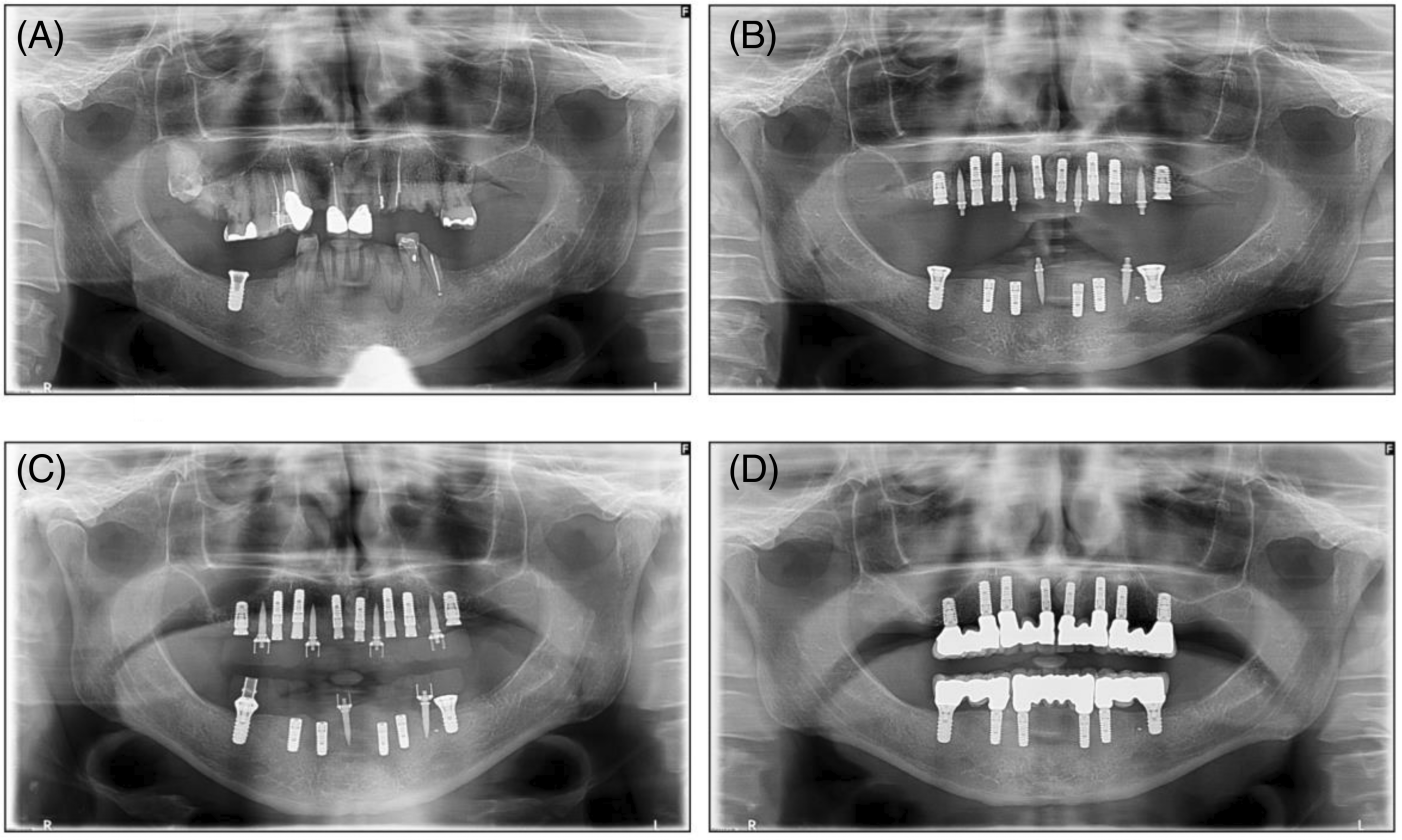
(A) Initial panoramic radiograph (pretreatment); (B) Position of regular implants and TIs postsurgery in the maxilla and mandible; (C) Loading of provisional prostheses onto maxillary TIs and onto mandibular TIs and one regular implant that was already in the jaw pretreatment; (D) Final fixed prostheses in the maxilla and mandible supported by regular implants.

An overview of the technique used is shown in Figures 1, 2, 3, 4.

### 2.5 Outcome variables

Two researchers (BBD, IP) retrieved the records and extracted data independently. The following data were extracted: (1) patient's age at TI placement; (2) gender; (3) presence of systemic disease; (4) smoking history; (5) type of TI; (6) position and dimensions of TI; (7) success supporting a surgical guide; (8) success supporting a provisional prosthesis; (9) TI failure; (10) dates of TI placement, loading of provisional prosthesis onto TIs, and removal of TIs; (11) timing of regular implant placement and loading.

Treatment success was defined as using healthy TIs to support a surgical guide and a provisional prosthesis until the regular implants could be conventionally loaded. The definition of a healthy TI included the absence of clinical signs of inflammation, bleeding or suppuration, increased PPD from a previous examination, or bone loss after initial bone remodeling.^23^

TI survival was assessed and defined as the TI being present in the oral cavity regardless of any biological and technical complications until it reached its desired functional endpoint (i.e., until the loading of regular implants). TI treatment failure was determined at the prosthesis level and defined as an inability of the provisional prosthesis to be retained on TIs or regular implants, due to mobility, resulting in the need to transition to an interim removable prosthesis.

### 2.6 Statistical analysis

Data were tabulated in an electronic spreadsheet (Microsoft Excel) which was used to calculate descriptive statistics. Descriptive data was reported as means and standard deviations for quantitative variables, while frequency distributions and cross‐tabulations were used for categorical variables. Kaplan–Meier analysis was performed to evaluate the cumulative survival rates. Both patient and implant units of analysis were used regarding the outcome variable. All data analyses were performed using a dedicated software (SPPS® 28.0, SPSS Inc., Chicago, IL, USA.).

## 3 RESULTS

### 3.1 Patient characteristics

From 27 initially identified patients, 26 were included in the study based on the eligibility criteria. One patient was excluded since the TIs were used to retain an overdenture. Eleven patients were female (42%), and 15 patients were male (58%). The mean patient age at TI placement was 61 (range: 42–81, SD = 9.51). The majority of the patients were nonsmokers (*n* = 17, 65%). The patient demographic data are reported in Table 1. When analyzing the maxillary and mandibular arches separately, there were 35 jaws since some individuals received TIs in both the maxilla and mandible.

**TABLE 1:** Patient demographics (*n* = 26).

Patient characteristic	*N* (%)
Gender	
Male	15 (57.7)
Female	11 (42.3)
Age	
40–50	5 (19.2)
51–60	5 (19.2)
≥61	16 (61.5)
Smoking status	
Nonsmoker	17 (65.4)
Ex‐smoker	5 (19.2)
Smoker ≤10/day	3 (11.5)
Smoker >10/day	1 (3.9)
Systemic disease	
Obesity (BMI > 25)	9 (34.6)
Diabetes (type 1 or 2)	5 (19.2)
Hypertension	10 (38.5)
Cardiovascular disease	3 (11.5)
Osteoporosis	1 (3.9)
Arthritis	2 (7.7)
No systemic disease	7 (26.9)
Other	8 (30.8)

### 3.2 Treatment success

**FIGURE 5:**
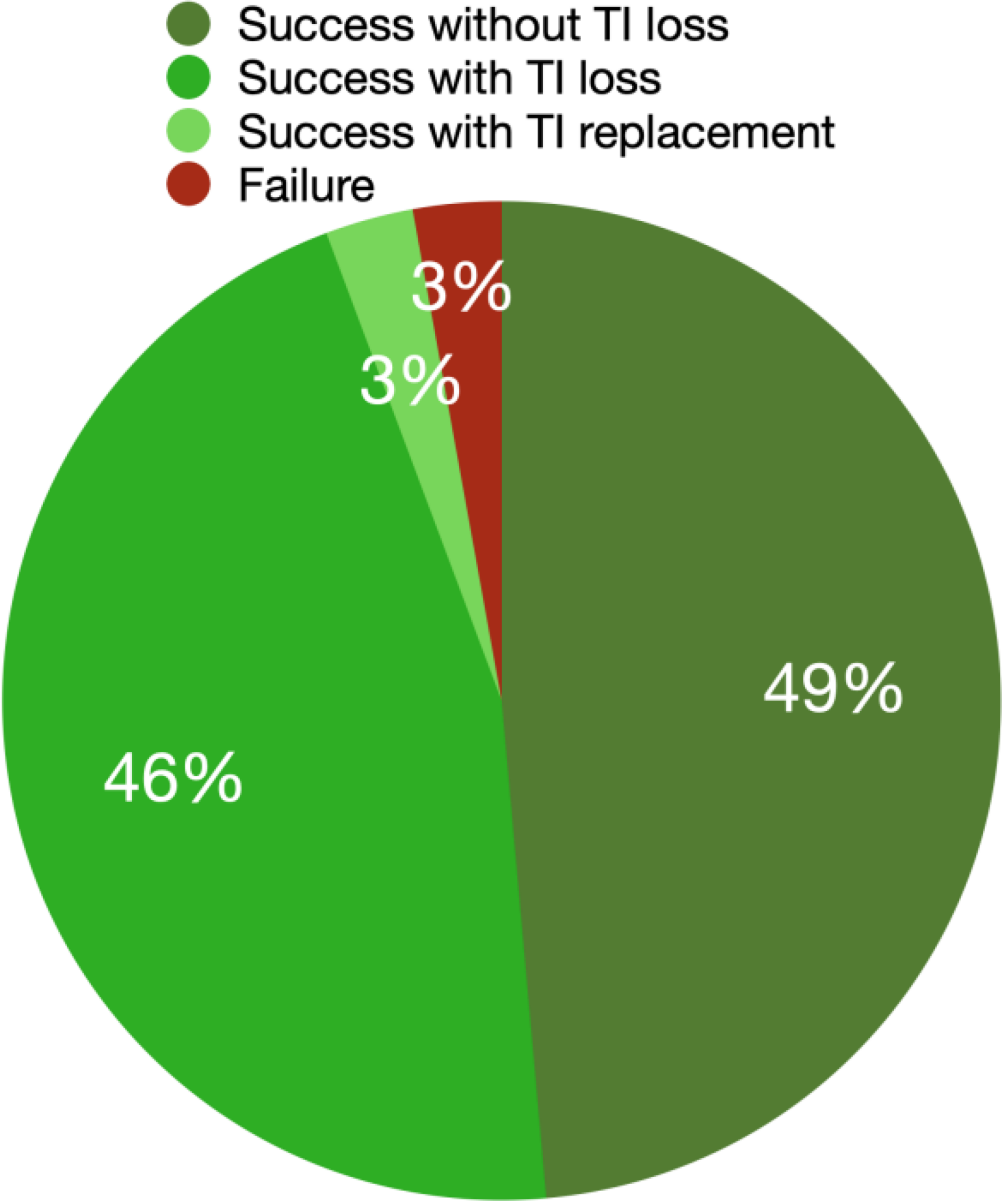
TI provisional prostheses support success distribution

The overall TI surgical guide support and provisional treatment success rate was 97%, either with or without the loss of individual TIs along the provisional stage period (Figure 5). In 17 out of 35 jaws (49%), no TIs were lost throughout the planned provisional period. In 16 jaws (46%), a loss of TIs occurred but had no impact on the treatment success since either the provisional prosthesis was able to be supported on the remaining TIs or the regular implants were already osseointegrated and could be loaded. In one jaw (3%), a TI was replaced during the provisional phase, allowing successful continued provisional prosthesis retention on TIs. Only one jaw (3%) had a loss of multiple TIs four weeks after implant placement surgery and an interim maxillary removable prosthesis was provided for the remainder of the healing period prior to successfully loading the regular implants.

### 3.3 Surgical guide function

**FIGURE 6:**
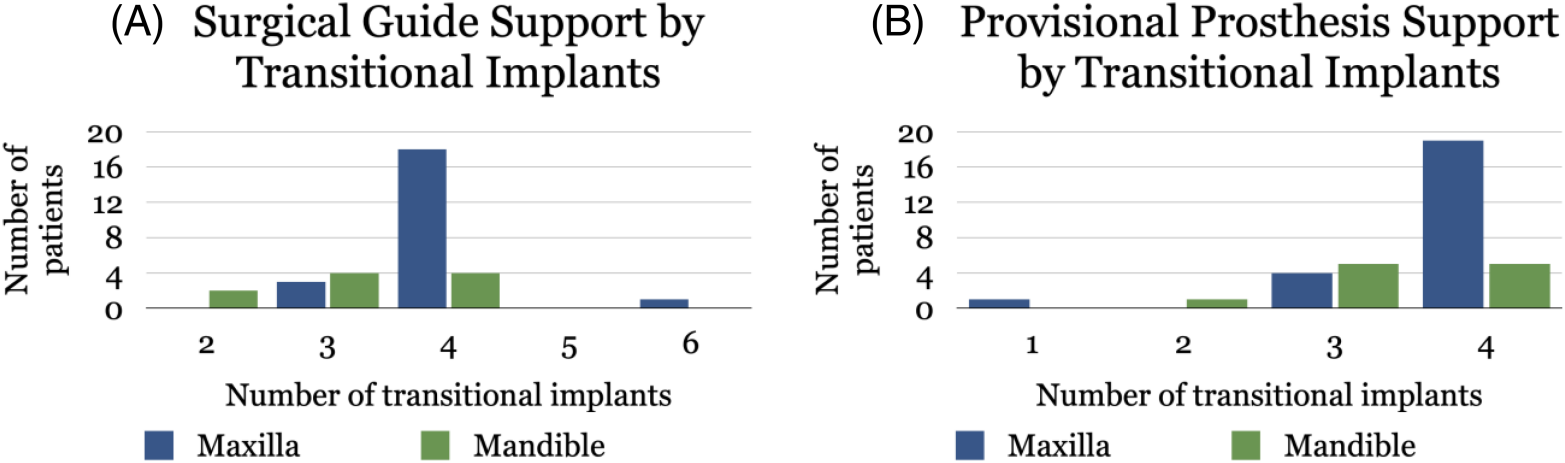
(A) Distribution of TI use for surgical guide support. (B) Distribution of TI use for provisional prosthesis support.

A total of 119 TIs were used to support surgical guides. TI‐supported guides were used in 22 patients for the maxilla, with an average of four TIs per patient. Guides were supported on three TIs in three patients (14%), four TIs in 18 patients (82%), and six TIs in one patient (4%). TI‐supported guides were used in 10 patients in the mandible, with an average of three TIs supporting the guide per patient. The guide was supported by two TIs in two patients (20%) and three or four TIs in four patients, respectively (40%), as shown in Figure 6A. One of the patients for whom the guide was supported by only two TIs already had a pre‐existing regular implant in their mandible, and therefore the guide was supported by both the TIs and the pre‐existing regular implant. The other such patient had a buccal perforation during the surgical placement of the third TI, preventing its placement, so the surgical guide was supported by only two TIs and some mobility of the guide was reported. Ninety‐four percent of TIs inserted before the regular implants proved suitable for supporting a surgical guide. The failure of TIs did not impact the overall use and fixation of surgical guides since the remaining TIs could be used as surgical guide support. One of the TIs (0.8%) intended for supporting a surgical guide was replaced before surgery due to mobility.

### 3.4 Provisional prosthesis support

Provisional prostheses were supported for a mean period of 6.5 months (SD = 6.86). On average, four TIs were used to support a provisional prosthesis. Twenty‐four patients received TI‐supported maxillary provisional prostheses. One patient had retention of a maxillary provisional using only one TI (4%) as several teeth were maintained during the provisionalization phase that was also loaded with the provisional prosthesis and subsequently extracted in a staged approach.^24^ Three TIs supported a maxillary prosthesis in four patients (17%) and four TIs did so in 19 patients (79%). Eleven patients received TI‐supported mandibular provisional prostheses. These were supported by two TIs in one patient (9%) and three or four TIs in five patients each (45%). The patient with the mandibular provisional supported by only two TIs is the same as the one for whom the surgical guide was also only supported by two TIs due to the presence of a previously placed implant that helped to support the surgical guide and provisional prosthesis. Figure 6B shows the distribution of TI use by jaw.

### 3.5 Transitional implant characteristics and survival

A total of 136 TIs were included in the study and 119 TIs were used to support surgical guides. Four remaining TIs failed before implant placement, 10 were placed after regular implant placement to support a provisional prosthesis, and three were inserted to replace failing TIs during the provisional phase.

**FIGURE 7:**
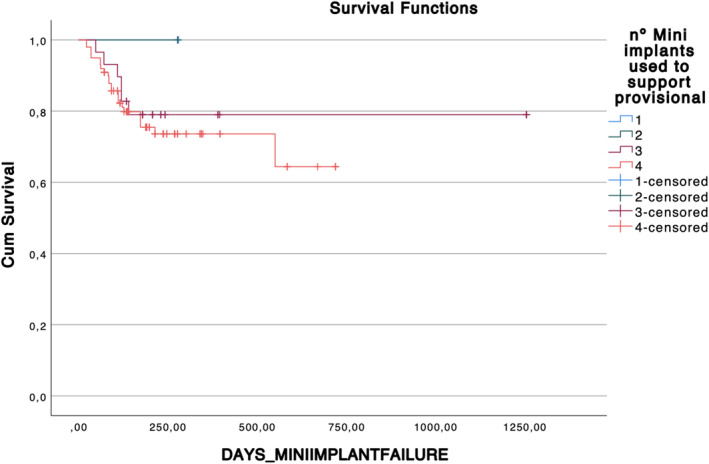
Kaplan‐Meier curve representing TI cumulative survival toward days of TI function depending on number of TIs supporting the provisional. Censored events represent the exit of the monitoring period due to definitive loading of the prosthesis and therefore the extraction of the TI.

The majority of TIs used allowed for a screw‐retained surgical guide and provisional prosthesis. Ninety‐six TIs were placed in the maxilla (71%) and 40 in the mandible (29%). Thirty‐five out of 136 TIs did not reach the removal point and failed at different stages of the provisional phase, yielding a survival rate of 74.26% (Figure 7). Eight of these 35 TIs demonstrated mobility at the time of surgery before loading the provisional, and 27 failed while supporting the provisional. The TI‐related variables are reported in Table 2.

**TABLE 2:** Transitional implant‐related variables (*n* = 136).

Implant variable	*N* (%)
Implant dimensions	*N* = 136
1.8 × 10 mm	53 (39.0)
1.8 × 14 mm	11 (8.1)
2.0 × 12 mm	2 (1.5)
2.2 × 7 mm	15 (11.0)
2.2 × 10 mm	28 (20.6)
2.2 × 12 mm	6 (4.4)
2.2 × 14 mm	17 (12.5)
2.4 × 10 mm	2 (1.5)
2.4 × 14 mm	2 (1.5)
Implant manufacturer	
ANEW	132 (97.1)
Zest	4 (2.9)
Type of bone	
1–2	19 (14.0)
2–3	68 (50.0)
3–4	49 (36.0)
Jaw	
Maxilla	96 (70.6)
Mandible	40 (29.4)
Transitional implant position	
3	1 (0.7)
4	20 (14.7)
5	5 (3.7)
6	2 (1.5)
7	20 (14.7)
8	2 (1.5)
9	2 (1.5)
10	19 (14.0)
11	2 (1.5)
12	5 (3.7)
13	18 (13.2)
18	4 (2.9)
20	7 (5.1)
23	6 (4.4)
24	4 (2.9)
25	5 (3.7)
26	3 (2.2)
27	1 (0.7)
29	7 (5.1)
31	3 (2.2)

The majority (*n* = 18, 51%) of the failed TIs had an implant diameter of 1.8 mm and a length of 10 mm, were placed in type three or four bone (*n* = 19, 54%), and were predominantly placed in the maxilla (*n* = 24, 69%). Regarding the implant position, most of the failed TIs (*n* = 10, 29%) were in the maxillary right first premolar position, followed by the maxillary left second premolar position (*n* = 5, 14%).

The TIs were predominantly inserted more than 24 h before regular implant placement (66%). Thirty‐one percent of TIs were placed on the same day as the regular implants, were picked up intraorally, and used to support a surgical guide and a provisional prosthesis, and 3% were placed after the regular implant placement to help support provisional prostheses.

### 3.6 Regular implant survival

Overall, 216 bone‐level implants were placed in 26 patients. Of these, 199 were placed in a fully guided TI‐supported manner, and four were placed using a tooth‐supported guide. Simultaneous bone augmentation procedures were performed in 22 patients. Of the 163 implants placed with GBR, there was a 98% survival rate, and three implants failed within six months after implant placement. All cases were managed without the use of bone reduction techniques.

## 4 DISCUSSION

The findings of this study, which examined 26 patients, provide valuable insights into the use of TIs in s‐CAIS and to support provisional prostheses.

The failure of TIs did not impact the overall use and fixation of surgical guides since the remaining TIs could be used as surgical guide support. Merely one of the TIs (0.8%) intended for supporting a surgical guide was replaced before surgery due to failure. Additionally, 94% of the TIs inserted before the regular implants were suitable for supporting a surgical guide. Most TIs that failed did so during the provisional phase, which did not have a detrimental impact on the treatment outcome. This was attributed to either the remaining TIs proving sufficient for supporting a provisional prosthesis or the completion of the healing period for the regular implants, which could then be utilized to also support the provisional prostheses. During the provisional phase, three failing TIs necessitated replacement to support a provisional prosthesis, and the failure of multiple TIs in one patient's maxilla resulted in the adoption of a removable prosthesis.

Guided implant placement, in general, has been shown to have multiple advantages compared to nonguided implant placement procedures in previous studies. These advantages include significantly increased precision, reduced complication risk during surgery, reduced risk of implant failure, and the allowance of a flapless surgical approach, thereby making the procedure less invasive.^7^, ^9^, ^10^, ^25^, ^26^ According to previous studies, implant‐ or tooth‐supported surgical guides exhibit higher accuracy than mucosa‐supported surgical guides.^11^, ^27^, ^28^ Therefore, for patients with failing dentition, it can be advantageous to retain three to four nonmovable teeth during the surgical and provisional phase to support a surgical guide and provisional prosthesis.^11^ When this is not feasible, or the patient is completely edentulous, the use of TIs to support surgical guides and to support provisional prostheses until the regular implants can be loaded with final restorations has been shown to have multiple advantages over other existing treatment strategies.^15^ TIs also facilitate flap elevation in cases where guided bone reconstruction is needed and provide greater access for irrigation and surgical visualization. This is a significant advantage compared to mucosa‐supported guides, where overheating of bone in flapless procedures can occur, and flap elevation leads to decreased guide support.^28^ This facilitates straight implant position, guided bone regeneration procedures, and submucosal healing of regular implants.

Surgical strategies to facilitate the immediate loading of dental implants in edentulous patients include the vertical reduction of bone height to a level where the alveolar bone is wider, and angulating implants to allow for increased lengths with higher primary stability.^29^ This can facilitate expedited treatment and increase prosthetic space; however, there are surgical risks and compromises in implant positions that can occur, as well as the loss of vestibular depth which can impede access for hygiene and maintenance. The use of TIs can reduce the need for bone reduction procedures, by facilitating the provision of regenerative solutions and maintenance of alveolar bone height together with a fixed provisional restoration. The use of TIs also allows for an ideal 3D implant position to be achieved based on the intended definitive restoration without using longer or wider implants or tilted implant positions in order to obtain higher insertion torque. In the cases included in the present study, immediate implant placement and bone augmentation procedures were employed whenever feasible. The choice of FP1 fixed implant‐supported prosthesis was pursued when specific criteria, such as transitional area, smile line, prosthetic volume, and site‐specific considerations assessed during the planning phase, allowed for its application.^30^ Bone regenerative strategies align with the fundamental principles of PASS, ensuring primary closure and space maintenance, in edentulous patients, even during the healing phase when provisional prostheses are often required.^31^ TIs enable the delivery of a fixed provisional prosthesis for the patient on the day of the surgery, improving their quality of life without interfering with bone augmentation procedures, as it ensures no pressure from the prosthesis to the jaw in augmented regions. The results of the present study further support this TI application, as they were used in most cases to support surgical guides and provisional prostheses until the regular implants could be loaded.

TIs have been previously used for other applications as well. TIs are effective alternative treatments to support a mandibular overdenture in patients with decreased bone volume in the anterior mandible.^32^, ^33^, ^34^, ^35^ A previous study reported implants <3 mm survival rate of 92% after up to seven years.^33^

In the present study, provisional prostheses were supported on TIs for a mean time of 6.5 months. Even though the regular implants could have been loaded earlier, different factors might have extended the provisional stage: (1) several patient cases were completed during the COVID‐19 pandemic, which caused delays in care, and (2) this study was conducted in an educational setting, where patient care is handled by residents, which can often result in extended treatment times due to resident graduation and turnover of care.

Results from this study should be interpreted cautiously, as due to its retrospective nature, data recovery from records has been limited. Also, presented data is mainly analyzed descriptively, and no direct comparison with other approaches than TI support was performed. Due to a convenience sample related to available patients, the sample size is limited, and so is the number of failures, hence no regression analysis of those failures could be performed. Another limitation of our study is associated with the restricted capabilities of digital tools 10 years ago, which necessitated the preplacement of TIs ahead of standard implant surgery. This process differs from the current protocol, wherein all procedures can be completed in a single appointment. Limitations associated with the use of TIs include high costs, since due to the placement of both TIs and regular implants, patients end up receiving more implants than they would without such a treatment protocol. Furthermore, in the case of total TI failure, patients will still have to receive a removable complete denture.

## 5 CONCLUSIONS

Using TIs to support a surgical guide and provisional prosthesis may present a valid treatment approach with high success rates:

- All implant surgical guides planned to be supported on TIs were successfully used this way.
- TIs could also support most fixed provisional prostheses until the regular implants could be loaded.

This technique holds several advantages:

- Allows flap elevation for bone reconstructive procedures without interfering with the surgical guide support.
- Provides a fixed implant surgical guide.
- Facilitates the delivery of a fixed provisional prosthesis on TIs regardless of the primary stability of the regular implants, which eliminates possible interferences of a removable prosthesis with augmented sites.

## AUTHOR CONTRIBUTIONS

Ignacio Pedrinaci (concept/design, data analysis/interpretation, drafting article, critical revision of article, data collection); Betty Ben‐Dor (data collection, data analysis/interpretation, drafting article, critical revision of article); Dominique Rousson (concept design, surgeries); Alejandro Lanis (drafting article, critical revision of article); Javier Sanz‐Esporrín (statistics, data analysis/interpretation, critical revision of article); Kevser Pala (data analysis/interpretation, drafting article, critical revision of article); German Gallucci (concept/design, data analysis/interpretation, drafting article, critical revision of article); Adam Hamilton (concept/design, data analysis/interpretation, drafting article, critical revision of article, approval of article).

## CONFLICT OF INTEREST STATEMENT

The authors declare no conflicts of interest.

## Data Availability

The data that support the findings of this study are available on request from the corresponding author. The data are not publicly available due to privacy or ethical restrictions.
